# Supplementary material for: Association between oral microbiome and seven types of cancers in East Asian population: a two-sample Mendelian randomization analysis
Source: Front Mol Biosci. 2023 Nov 21;10:1327893. doi: 10.3389/fmolb.2023.1327893 (PMC10702768; doi:10.3389/fmolb.2023.1327893)
Supplement: Supplementary file 1 [file DataSheet1.ZIP › Supplementary Materials/MR plots of saliva/saliva═╝/Lung Cancer/pheno.2919_to_lung cancer_leave_one_out.pdf]

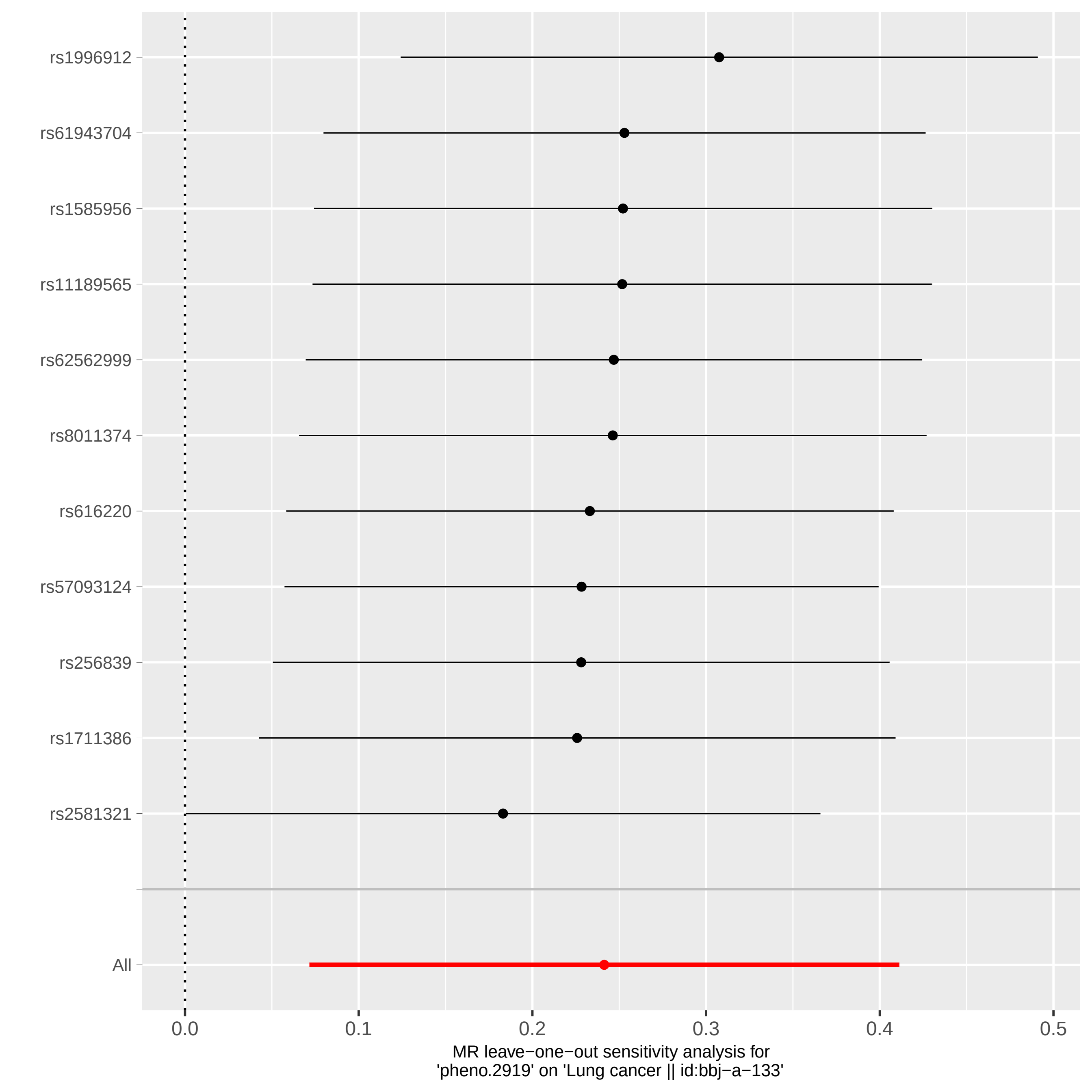

rs1996912
rs61943704
rs1585956
rs11189565
rs62562999
rs8011374
rs616220
rs57093124
rs256839
rs1711386
rs2581321
All
0.0
0.1
0.2
0.3
0.4
0.5
MR leave−one−out sensitivity analysis for
'pheno.2919' on 'Lung cancer || id:bbj−a−133'
